# Human genotype-to-phenotype predictions: Boosting accuracy with nonlinear models

**DOI:** 10.1371/journal.pone.0273293

**Published:** 2022-08-31

**Authors:** Aleksandr Medvedev, Satyarth Mishra Sharma, Evgenii Tsatsorin, Elena Nabieva, Dmitry Yarotsky

**Affiliations:** Skolkovo Institute of Science and Technology, Moscow, Russia; Wright State University, UNITED STATES

## Abstract

Genotype-to-phenotype prediction is a central problem of human genetics. In recent years, it has become possible to construct complex predictive models for phenotypes, thanks to the availability of large genome data sets as well as efficient and scalable machine learning tools. In this paper, we make a threefold contribution to this problem. First, we ask if state-of-the-art nonlinear predictive models, such as boosted decision trees, can be more efficient for phenotype prediction than conventional linear models. We find that this is indeed the case if model features include a sufficiently rich set of covariates, but probably not otherwise. Second, we ask if the conventional selection of single nucleotide polymorphisms (SNPs) by genome wide association studies (GWAS) can be replaced by a more efficient procedure, taking into account information in previously selected SNPs. We propose such a procedure, based on a sequential feature importance estimation with decision trees, and show that this approach indeed produced informative SNP sets that are much more compact than when selected with GWAS. Finally, we show that the highest prediction accuracy can ultimately be achieved by ensembling individual linear and nonlinear models. To the best of our knowledge, for some of the phenotypes that we consider (asthma, hypothyroidism), our results are a new state-of-the-art.

## Introduction

The problem of predicting phenotype from genotype is a “holy grail” of modern genetics, with practical applications in fields such as personalized medicine [1] and genomic selection for agriculture [2], and is an active area of research. Its relevance has grown with the affordability of genotyping, and will likely continue to increase as sequencing becomes more commonplace.

Classical predictive models for human phenotypes from full genotypes are variations of linear methods; a popular approach is to use a method that combines regression with suitable regularization (e.g., Lasso [3]). Due to the size of the data and computational limitations, regression is typically preceded by feature selection through genome-wide association study (GWAS) methods which identify the features (Single Nucleotide Polymorphisms, SNPs) most significantly associated with the phenotype, although methods that do not always need this step are being developed [4, 5]. Linear models work well with the large number of SNPs, many of which have small effects, and with the limited amount of data (available genotype-phenotype pairs). In a striking success of linear methods in the genotype-to-phenotype prediction problem, polygenic risk scores that are computed on the basis of a person’s entire genotype, have been shown to be as well or more sensitive in identifying individuals susceptible to certain diseases as Mendelian risk genes [6].

At the same time, non-linear effects, such as epistasis, can be a significant factor contributing to a phenotype [7]; consequently, there are indications that models which account for non-linearity, for example, for interactions between features, can have better prediction accuracy and may alleviate the problem of lack of the generalizability of genotype-based predictions across genetic backgrounds, at least on simulated data and/or model organisms [8, 9].

If nonlinear effects do indeed contribute significantly to some phenotypes, then latest-generation machine learning methods, which take nonlinearity into account, should outperform linear ones. These methods require large amounts of training data, which is now becoming available through datasets encompassing hundreds of thousands of individuals, such as the UK Biobank [10]. So far, the application of nonlinear machine-learning methods to the phenotype prediction problem has been inconclusive [11–13]. For some phenotypes, the effects may indeed be overwhelmingly additive and nonlinear methods may not contribute much (height is believed to be one such example [14]), while others may have a genetic architecture involving interactions both among genotypes and between genotypes and other covariates such as age.

This discussion motivates three questions that we address in this paper.

**Nonlinearity**: Is there any nonlinearity in the human genotype-phenotype relationship that can be efficiently exploited by state-of-the-art machine learning methods to reliably improve prediction accuracy?
**SNP selection**: How optimal is the state-of-the-art pipeline of GWAS-based SNP selection followed by a predictive model? Can it be improved by incorporating the nonlinearity into the selection step of the prediction pipeline?
**Most accurate models**: What are, ultimately, the most accurate models for human genotype-to-phenotype prediction? How much of an improvement over the current state of the art can we achieve by using nonlinear methods, adjusting the pipeline, and aggregating results of different methods?

## Our contribution

We address the above questions by considering several commonly studied human phenotypes and systematically exploring how the prediction accuracy is affected by the model type (linear or gradient boosted decision trees), the strategy of feature selection, and the strategy of aggregating (ensembling) optimal individual models.

**Nonlinearity**: Our main tool in examining nonlinearity is experiments with gradient boosted decision trees of different depth, as implemented by the library XGBoost [15]. Trees of depth 1 depend linearly on SNPs, while deeper trees depend on them nonlinearly, which allows us to directly compare models with or without nonlinearity (see Section XGBoost). In addition, we compare these results with the performance of the state-of-the-art linear method, Snpnet [4]. Moreover, we analyze the significance of nonlinear effects with respect to the number of additional (non-SNP) covariates included in the model, and with respect to interactions between features within the same group or between different groups (SNPs, covariates).

**SNP selection**: We propose a new approach to SNP selection, based on constructing a preliminary lightweight XGBoost model (see Section SNP selection by XGBoost). Our approach selects different set of SNPs which is 2–5 times smaller than GWAS but achieves a similar prediction accuracy.

**Most accurate models**: We identify the top performing models constructed by individual algorithms such as XGBoost and Snpnet, and combine them to further improve the prediction accuracy. As usual in machine learning, the most accurate models are obtained by ensembling a number of (preferably, sufficiently diverse) more simple models. We consider several ensembling strategies (see Section Ensembling and stacking).

## Methods

### Lasso and Snpnet

Most current methods for the prediction of phenotype from genotype are based on some form of penalized or Bayesian regression [16]. Lasso [3], which is linear regression with an *ℓ*_1_-norm penalty, is well-suited for this task as genotype matrices are compressed sensors [17] and are sparse with respect to almost any phenotype. Lasso uses an absolute value regularization of parameters and tends to drive most of them to zero, thus selecting only a relatively small number of features.

Snpnet is a modification of Lasso that enables finding exact solutions to extremely high dimensional multivariate regression tasks on large datasets through an iterative batched screening process [4]. As Snpnet achieves state of the art performance in the prediction of several phenotypes, we opted to choose it as a baseline linear method to compare the performance of nonlinear methods to.

### XGBoost

**Fig 1:**
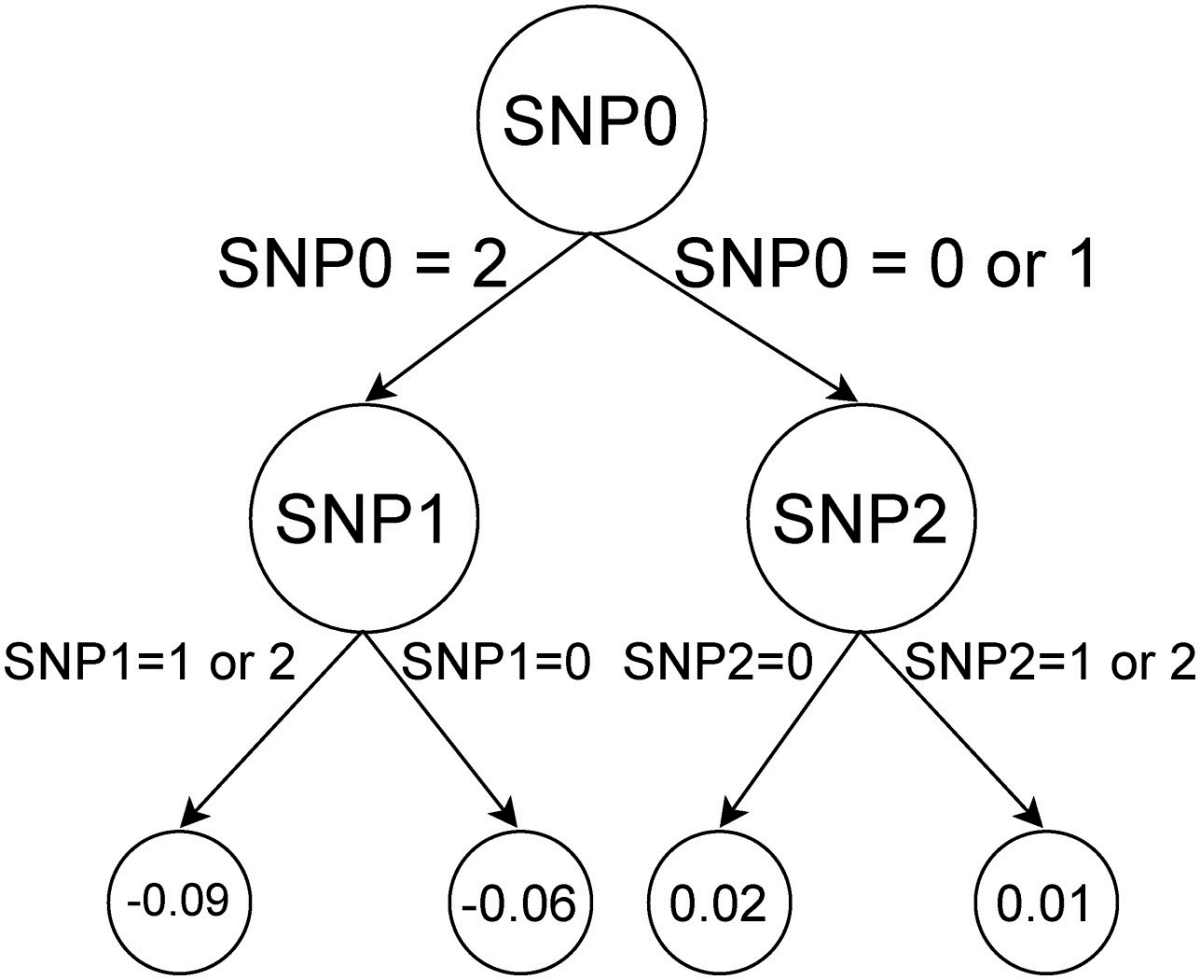
Example of a depth-2 decision tree that uses three different SNPs. Leaf nodes represent the weights that these particular SNP values contribute to the value of final prediction for a single datapoint. The genotypes are encoded as 0: homozygous reference, 1: heterozygote, 2: homozygous alternative.

XGBoost (for “eXtreme Gradient Boosting”) [15] is a well-known implementation of gradient boosted decision trees [18]. XGBoost iteratively adds trees to the model by optimizing the following objective at each step *t*:

$$\begin{array}{c} L^{\left(t\right)}=\sum_{i=1}^{n}l(y\left(i\right),{\hat{y}}^{\left(t-1\right)}\left(i\right)+f_{t}\left(x\left(i\right)\right)+\Sigma \left(f_{t}\right), \end{array}$$

where **x**(*i*) is the input feature vector for the instance *i*, ${\hat{y}}_{i}^{t}$ is the prediction for the instance *i* at step *t*, *l* is the loss function, *f*_*t*_ is the new decision tree, *f*_*t*_(**x**_*i*_) are the new tree predictions and Σ(*f*_*t*_) is a penalization term for the complexity of the new tree. The full XGBoost model is the sum of all the constructed decision trees. Each split in the tree is performed by comparing one of the features to a threshold value. We can interpret a learned XGBoost model by inspecting the trees it consists of and the features they split on, with each feature being a particular SNP or covariate. An example of such a tree with depth 2 is shown in Fig 1. Values in leaf nodes represent the influence of these particular combination of SNPs and their values on the phenotype.

It is important to note that an XGBoost model of depth 1 is a sum of *univariate* functions (i.e., depending on a single input feature *x*_*k*_, e.g., one SNP). Conversely, any sum of univariate functions can be represented (or approximated, in the case of continuous variables) by a depth-1 XGBoost model. (This is so because any univariate function *f*(*x*) can be represented or approximated by linear combinations of the indicator functions **1**{*x* > *c*} with various thresholds *c*.) In this sense, depth-1 XGBoost models are equivalent to *linear* models defined on one-hot-encoded input variables. For example, if different features ${\left(x_{k}\right)}_{k=1}^{K}$ represent *K* SNPs with values 0,1,2, then a general depth-1 XGBoost model on these features is equivalent to the linear model

$$\begin{array}{c} \hat{y}=\sum_{k=1}^{K}\sum_{j=0}^{2}\beta_{kj}1\left\{x_{k}=j\right\} \end{array}$$

with arbitrary coefficients *β*_*kj*_.

In contrast, XGBoost models of depth 2 or higher are able to consider pairwise interactions between different features. It is generally expected that a part of phenotype variance is due to epistatic interactions between SNPs [19]; also, there might be interactions involving environmental features (covariates) used in the prediction. Accordingly, we can test how much predictions benefit from including pairwise interactions by comparing depth-1 and higher-depth XGBoost models. In this work we consider only depths 1 and 2, because larger depths do not bring noticeable improvement on our data.

XGBoost can naturally incorporate some pairwise epistatic interactions of SNPs by placing them in one tree. For example, in Fig 1 it can assign any effect sizes to the combinations of (SNP0 = 2 and SNP1 = 1 or 2) and (SNP0 = 2 and SNP1 = 0). If there is an epistasis in the form *y* = *β*_*ep*_ ⋅ *I*_*SNP*0=2_ ⋅ *I*_*SNP*1=0_ (where *I* denotes an indicator variable), then the XGBoost tree in Fig 1 can catch it. In this case, *β*_*ep*_ is the effect of interaction between SNP0 = 2 and SNP1 = 0 on phenotype *y*.

A useful feature of XGBoost is its ability to restrict interaction between sets of features in single trees and thus enable the study of feature interactions. By allowing or disallowing XGBoost to combine genotype and environment features in the same trees, we can test the effects of single SNP–single environment feature interactions while controlling for model expressivity in other respects.

Another useful feature of XGBoost is the reporting of feature importance scores, which is used in the XGBoost-based SNP selection approach that we propose in this paper.

An important advantage of XGBoost over more complex methods such as neural networks is that it has only a small number of important hyperparameters and is accordingly easy to tune. One of these hyperparameters is the tree depth; as already noted, we set it to 1 or 2. Another important hyperparameter is the number of trees; in our experiments we monitor the XGBoost performance throughout learning and select the optimal number of trees. We observe the other hyperparameters to provide the optimal performance at their default values, except the *l*_1_ regularization parameter *α* as in Lasso. We use *α* from 15 to 20 for all our models.

Conversely, XGBoost is a more complex method compared to regularized linear models and can be considered to be less interpretable. While several techniques for interpreting black-box machine learning models in general [20] and XGBoost specifically [21] exist, this is an important consideration in clinical applications in personalized medicine and can be a potential barrier to adoption [22].

### Ensembling and stacking

We can combine predictions obtained from several different models in order to obtain a more accurate predictor that may mitigate the shortcomings of each individual model by averaging their biases. This is a commonly used technique in machine learning called ensembling [23]. We investigated how constructing an ensemble of predictions from different models can be used to improve the overall prediction accuracy.

We considered several ensembling strategies starting with a simple ensemble with unweighted averaging of the predictions of the individual models, which shows a modest but consistent improvement in performance. This strategy proves to be less effective when the predictive models show significantly different performances. In this latter case we employ weighted averaging of predictions, with weights empirically estimated by maximizing the accuracy of the ensemble on a validation set. This approach can be viewed as a basic example of “stacking” [24] (i.e., a collection of initial models is “stacked” with a subsequently learned linear model).

One can consider more complex stacking strategies, aggregating predictions of initial models in a nonlinear way while also possibly taking additional covariates into account. In our experiments, however, these complex stacking strategies did not outperform the simpler models, and notably added additional complexity in terms of hyperparameter selection. For this reason, we limited ourselves to the unweighted and weighted averaging ensembles as described above.

### SNP selection by XGBoost

Addressing the question of optimizing the data processing pipeline, in this work we propose a new SNP selection method based on XGBoost as an alternative to GWAS-based SNP selection. A typical human genotype dataset has at least 600K called SNPs, and in many cases up to 90 million imputed SNPs. Moreover, UK Biobank recently released 200K exomes with more than 10 million SNPs [25].

The problem is that machine learning prediction models are not suited for handling millions of features without some feature selection. For example, phenotype prediction models typically use up to 10–100K SNPs, and these SNPs are preselected by GWAS. The largest phenotype prediction model available is Snpnet [4], which is able to use roughly 650K SNPs as features for the full UK Biobank dataset.

A drawback of GWAS-based SNP selection, however, is that the SNPs are selected based solely on their individual p-values, regardless of how much information an SNP brings compared to the other SNPs. As a result, we can expect SNPs strongly correlated with the predicted phenotype to be overrepresented in the selected subset, and the weakly correlated SNPs to be underrepresented. This is exactly the issue that we address.

**Fig 2:**
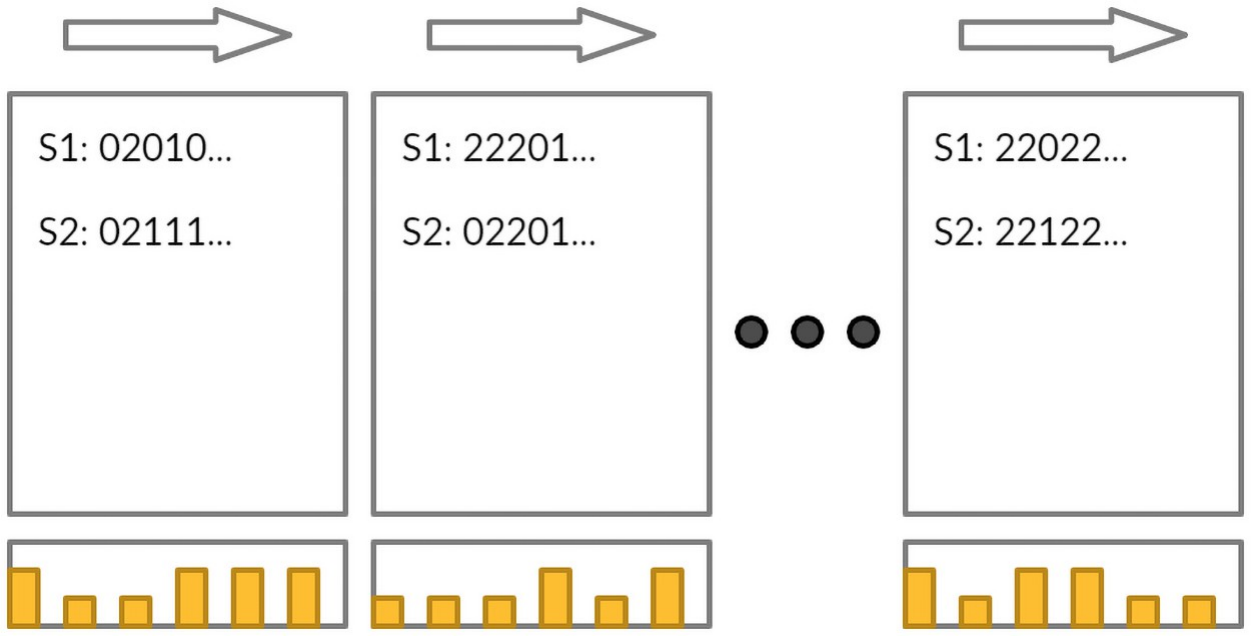
The iterative scheme of our XGBoost selection algorithm. Yellow bars are individual SNP importances for each window. S1, S2 are individual samples. *F*_*n*_ is the *n*’th XGBoost model, using the respective window of features. *F*_*n*_ model is fitted on predictions from model *F*_*n*−1_.

Our XGBoost-based pipeline selects a balanced, compact and mutually uncorrelated subset of relevant SNPs from the whole available dataset. For that, we split the sequence of all SNPs into multiple disjoint windows. Then, we fit a separate XGBoost prediction model *F*_*n*_ on each window *n* moving either from chromosome 1 to X or from X to 1. In each window, we calculate XGBoost feature importance scores and use them for the final SNP selection. Also, every new XGBoost window model uses predictions from the previous window as the starting point: it helps the model to select new SNPs uncorrelated with SNPs selected earlier. See Fig 2 for an illustration. In the case of UK Biobank data with the total of 700K SNPs, we use windows of size 10K.

We chose XGBoost instead of LightGBM [26] or CatBoost [27] because of their performance on prediction tasks. We selected 1K SNPs using GWAS for height, and trained XGBoost, LightGBM and CatBoost models. XGBoost was the best with test *r*^2^ = 0.613, LightGBM has *r*^2^ = 0.583 and CatBoost has *r*^2^ = 0.607. We decided to continue using XGBoost for subsequent experiments.

#### Simulated phenotypes

To assess the performance of our XGBoost selection (namely, its ability to detect epistatic SNPs, to catch SNP-environment interactions, and to work with the nonlinearity of the environment), we performed a number of experiments with simulated phenotypes, in addition to the main set of experiments with actual phenotypes. A simulated phenotype is modelled as a weighted linear combination of linear effects **y**_*l*_, pairwise epistatic effects **y**_*ep*_ and random noise **y**_*ϵ*_:

$$\begin{array}{c} y=w_{l}y_{l}+w_{ep}y_{ep}+w_{\epsilon }y_{\epsilon } \end{array}$$

Here, **y**, **y**_*l*_, **y**_*ep*_, **y**_*ϵ*_ are sample-dependent, while *w*_*l*_, *w*_*ep*_, *w*_*ϵ*_ are sample-independent weights that sum to 1 and are used to adjust the relative contribution of different terms. The linear effects **y**_*l*_ linearly depend on individual SNPs, while the epistatic effects **y**_*ep*_ are formed by events involving random pairs of SNPs. See Section for simulation details.

### Metrics and error estimation

We predict several numerical as well as categorical (binary) phenotypes, and assess their accuracy using the standard *r*^2^ (coefficient of determination) and ROC AUC (area under the receiving operator characteristic curve) metrics, respectively. Since our models have accuracy close to the state-of-the-art, it is important to carefully estimate the standard errors of these metrics. We use two approaches for that: one based on subsampling and another on the explicit form of the ROC AUC statistic (for categorical phenotypes). The two approaches produce close values of standard errors. See S1 Appendix for details.

### Software and performance

We used XGBoost Python package of version 1.1.0 [15] for Python 3.6 and Snpnet version 0.3.0 for R 4.0.2.

The XGBoost package has GPU support and works well with missing data [15]. We built XGBoost models using one GPU node on a cluster with Nvidia Tesla V100 16GB and up to 200GB RAM [28]. In our experiments, XGBoost on this GPU was 3–6 times faster than on a CPU with 24 CPU cores. Training a single XGBoost model on GPU with 10K SNPs and 350K samples requires roughly 15GB of GPU memory and up to 80GB of RAM. The XGBoost selection step takes 1.5 hours, while building the final prediction model takes 2–3 hours. In comparison, GWAS takes 10–15 minutes on 24 CPU cores, and Snpnet requires up to 1.5 hours on 24 CPU cores on the same data.

## Data

### Dataset and preprocessing

The genotype and phenotype data for our experiments were obtained from the full release of the UK Biobank [10]. We selected a cohort of 429,351 individuals of white British ethnicity according to data-field 21000 ‘Ethnic background’ of the biobank so as to maintain a homogeneous population structure as in [4, 5, 29, 30]. This cohort was further subdivided into train, validation, and test sets. Our train set has 343,481 samples, validation and test sets have 42,935 samples each. A total of 701,347 variants remained after filtration for allele frequency with a cutoff of 0.5%. We opted not to filter variants by linkage disequilibrium as our methods can handle its presence. For each phenotype, variant selection by GWAS was performed using PLINK 2.0 [31] taking into account age, sex, and 10 genotypic principal components. Principal components were calculated with FlashPCA [32] using the training dataset exclusively. For PCA calculation we filtered SNPs by linkage disequilibrium as recommended by the FlashPCA authors. We did not use the principal components provided by the UK Biobank because those components are based on the entire dataset and therefore would violate the training vs validation vs hold-out dataset split.

#### Phenotypes

We selected two continuous and three binary phenotypes for our analysis: height, eBMD (estimated heel-bone mineral density), asthma, hypothyroidism and psoriasis. Height is known to be highly heritable [33], and it was predicted with high accuracy by linear models in [4, 34]. eBMD also has a relatively high heritability [35, 36] and there are efforts to predict it in [29, 37] with up to *r*^2^ = 0.25 and *r*^2^ = 0.12. Asthma and hypothyroidism have a high prevalence (11% and 5% in the UK Biobank data) and are predicted in [4, 30]. Psoriasis was selected to check how our methods work with a highly imbalanced phenotype (1% prevalence).

Only eBMD was preprocessed before prediction, using the same procedure as in [29, 37]. Additional 27 features for the eBMD prediction were selected using osteoporosis risk factors, because osteoporosis is defined as low eBMD value. These risk factors include age, sex, physical activity and alcohol consumption data, with the full list provided in S4 Table. Asthma, psoriasis and hypothyroidism were taken from the “Non-cancer illness code, self-reported” field 20002 of the UK Biobank main dataset. For each of three categorical phenotypes, we selected age, sex and 18 additional features. The resulting set of features is different for different phenotype. We did not select any additional features besides age and sex for height, because height is predicted very well from genotype data. Also, for each phenotype we added the same 10 leading principal components calculated using FlashPCA on train dataset.

## Results and discussion

### Individual models and phenotypes

We built three groups of models for each phenotype. The first group of models uses only genetic data and sex as a covariate (because sex is also a genetic feature). The second group uses sex, age and top 10 principal components from the genotype matrix built on the training set. The third group is our attempt to make the best possible prediction and find out some nonlinear dependencies between genotype and environment, and environment and phenotype. Models of this group use age, sex and 19 additional covariates (for asthma, psoriasis and hypothyroidism) or 27 covariates (for eBMD). We chose not to create the third group for height because it is too hard to choose a set of covariates without strong two-way interaction.

**Fig 3:**
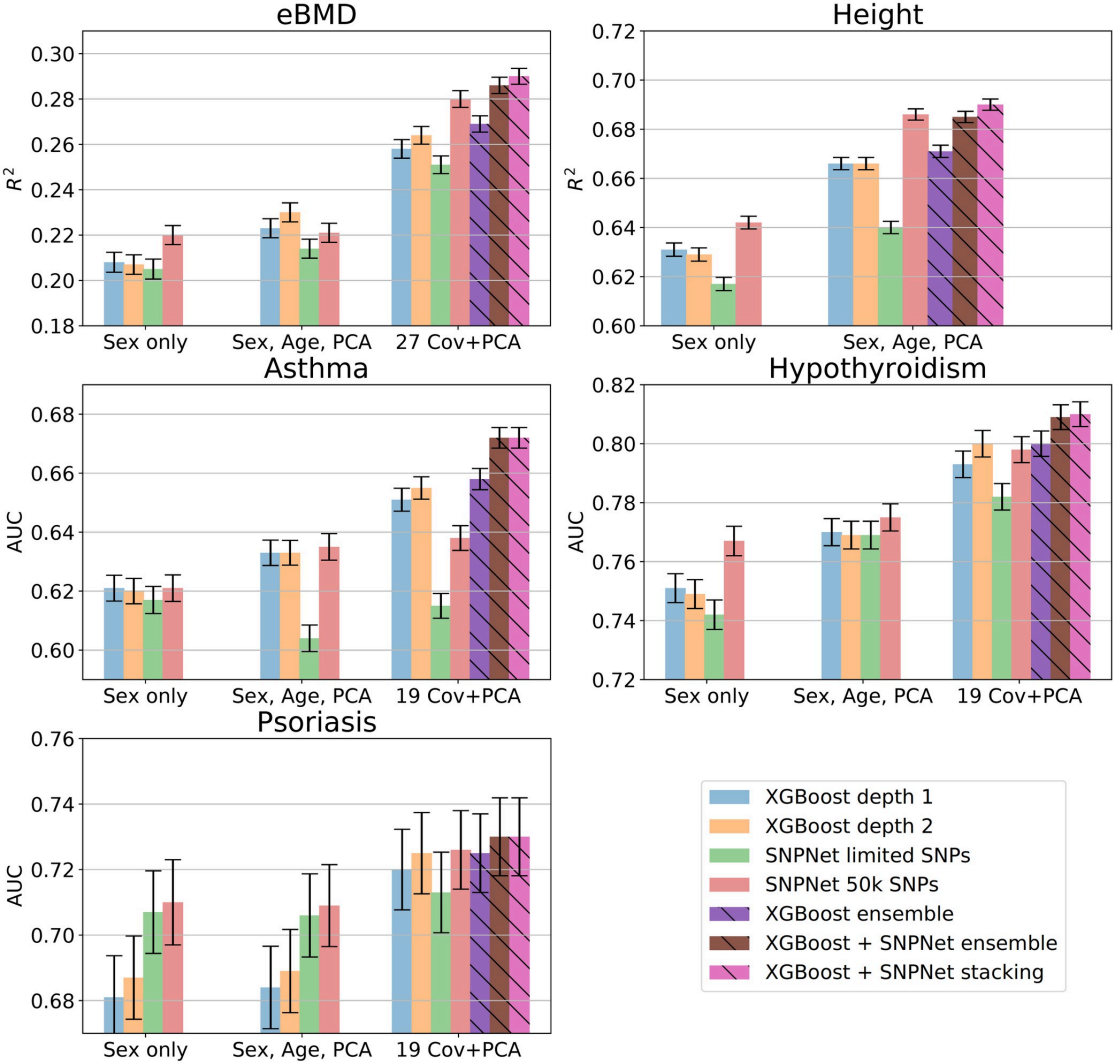
Prediction performance for all five phenotypes. Performance metrics (*r*^2^, AUC) and their standard deviations are computed on an independent test set. The total height of error bars is two standard deviations.

Each group includes four “individual” models, and the last group additionally includes two ensemble models built using the individual models of this group. Out of the four individual models, the first two are XGBoost models of depth 1 and 2. They are trained on the set of SNPs selected by XGBoost as described in section SNP selection by XGBoost. The other two individual models are linear Lasso models built by Snpnet on the set of GWAS–detected SNPs. One of them uses the same number of SNPs as the XGBoost models to demonstrate that XGBoost selection–based models need fewer SNPs than GWAS-based SNPs. The last Snpnet model is trained without limiting the number of SNPs. However, while Snpnet is quite scalable, we still found it difficult to train it on more than 50K SNPs on our hardware, so the number of SNPs for this model is 50K (in most cases) or 20K (if the 50K model does not display any AUC or *r*^2^ improvement over 20K). Fig 3 shows an overview of our results. See S2 and S3 Tables for numerical values.

#### Asthma

For asthma, results of the four individual models were similar in each of the three groups. We observe almost no difference between XGBoost depth 1 and depth 2, meaning that the influence of epistatic interactions is very small or non-existent in SNPs selected by XGBoost. The results in the second group show a similar picture. However, this is not the case for the last group, where XGBoost significantly outperforms GWAS-based Snpnet. Constructing ensemble of two XGBoost depth 2 models based on forward and backward SNP selection and one Snpnet model yields the best result for asthma: AUC 0.67. It demonstrates the utility of XGBoost models for asthma but Snpnet still helps us to get a better ensemble. This result is the best known to us for asthma, although every other attempt of predicting asthma in literature uses their own scheme of splitting and filtering the data. For example, Qian *et al*. [4] obtained an AUC of 0.613 using Snpnet, however they did not use any covariates other than age, sex, and principal components of the genotype matrix. Lello *et al*. [30] report an AUC of 0.632, but on genetic data only and on a test set composed of self-reported white but non-genetically British individuals from the UK Biobank.

#### Hypothyroidism

Results for Hypothyroidism are generally different from asthma. For genetic data only, Snpnet outperforms both XGBoost models. Adding age and 10 principal components allows XGBoost model to catch up with Snpnet. XGBoost starts to outperform Snpnet with a limited number of SNPs only when adding 19 more covariates. The ensemble is still the best model and has an AUC of 0.807, which is also the best result known to us up to this date. Lello *et al*. [30] report an AUC of 0.705, however the experimental set-up differs as they use imputed genotype data only to train their model. The discussion of different splits and filtering criteria is still applicable here. Another interesting moment is that XGBoost of depth 2 outperforms XGBoost depth 1 model with full set of covariates. That could mean that there are some nonlinearities in genotype-environment or environment-phenotype interactions.

#### Psoriasis

Psoriasis is a highly unbalanced phenotype with prevalence roughly 1.1%. For such an unbalanced phenotype AUC can be high even for a classifier with low precision.

Also, a large change in the number of false positives can lead to a small change in the false positive rate used in ROC analysis. That’s why our AUC std estimation gives a rather high value in this case, see S1 Table. Snpnet is still better than XGBoost for small number of covariates, but generally these results are inconclusive. Ensembles are slightly better than individual models, but the improvement is insignificant (0.1 − 0.2 standard deviations).

#### eBMD

For eBMD, the unlimited version of Snpnet is generally better than XGBoost in all three groups. XGBoost of depth 2 is better than depth 1 for data with covariates. This also suggests some nonlinearity related to environment. Although Snpnet works significantly better for 27 covariates, adding XGBoost models to the ensemble further improves the result, to *r*^2^ = 0.286. This result is also state-of-the-art, to the best of our knowledge. The best eBMD prediction is in [29], *r*^2^ = 0.246.

#### Height

Height is the well-known almost linear phenotype which is easy to predict. Our results generally agree with this expectation. XGBoost of depth 1 is even a bit better for genotype-only data. On the height data, XGBoost selection demonstrates its power to select a smaller set of SNPs (10K) which gives the same prediction power as bigger GWAS sets. Ensembles are still the best. That means that XGBoost and Snpnet recover slightly different prediction models.

Our result of 0.676 for the Snpnet is slightly worse than the one reported in the Snpnet paper [4]. This can be attributed to the fact that they used 650K genotyped SNPs without any GWAS preselection step, and that height is a highly additive phenotype. Also, the authors of Snpnet used a slightly different procedure of data splitting and filtering.

### Ensembles

**Fig 4:**
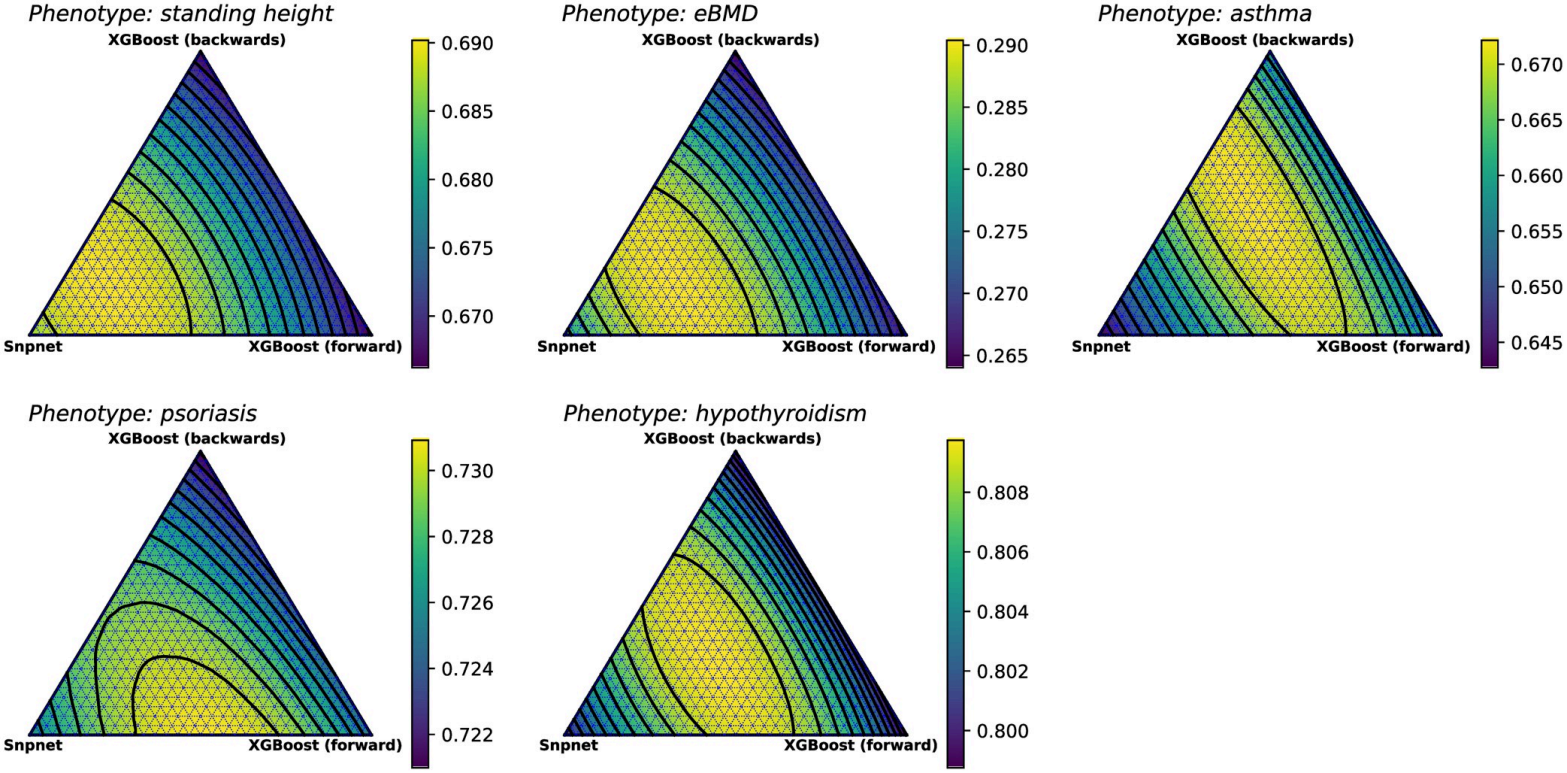
Simplex plots showing the metrics obtained by taking weighted sums from three prediction methods: Snpnet trained on 50k GWAS-selected SNPs, and XGBoost trained on XGBoost selected SNPs (forward and backward passes). The XGBoost models were trained on 10k SNPs for standing height and eBMD, 5k SNPs for psoriasis and hypothyroidism, and 1k SNPs for asthma. Each vertex of a triangle represents the performance of an individual model, with the center of the triangle representing the performance of the unweighted average.

We constructed ensembles using three individual models for each phenotype: the best performing Snpnet model on GWAS selected SNPs, and two separate XGBoost models trained on SNPs selected by XGBoost during forward and backward passes along the genome. These ensembles generate a final prediction based on a weighted sum of the predictions of each individual model. Fig 4 shows a simplex plot in which we see how the test metric changes with varying weights assigned to each model—the weights of ensembled models correspond to the barycentric coordinates of a point in the simplex.

As already mentioned, we consider two ensembling strategies—an unweighted average of the predictions from the constituent models (corresponding to the simplex center), and a weighted ensemble, where suitable weights are obtained by maximizing the metric for that phenotype on the validation set. The weighted ensemble shows a noticeable improvement in the test metric over the best individual model in all but one phenotype. Weighted ensembles always performs as well as or better than the simple ensemble, especially in cases where one method significantly outperforms the rest and the optimal point deviate significantly from the center of the simplex (for example, height in Fig 4).

### Analysis of nonlinearity

One can think of nonlinearity in the phenotype-to-phenotype dependence as resulting from three possible kinds of feature interactions: pure gene interactions, gene–covariate interactions, and pure covariate interactions. Since some of the covariates are numerical and so potentially carry complex information, the last group may also include “self-interactions”, in the sense that a phenotype may depend nonlinearly on a particular single covariate. We analyze the three interaction groups one-by-one.

**Pure gene interactions**: This type of interactions should manifest itself in a better performance of the depth-2 XGBoost models compared to the depth-1 models on data not involving additional covariates, i. e. in our ‘Sex-only’ group of results. However, as wee see in Fig 3, the differences between these two models are insignificant in this group for all the considered phenotypes. It is important to note that depth-2 XGBoost model is well suited only for pairwise interactions and not for higher-order ones. Therefore it remains possible that the gene interactions for these phenotypes are high-order.

**Gene–covariate interactions**: The presence of these interactions can be tested by restricting the XGBoost trees to contain either only SNP features or only covariate features (since in this case the model becomes a sum of two terms—one depending on the SNPs and another on the covariates). In Table 1 we compare models trained with or without this restriction. We see that there is almost no difference between such models (and even if present, it is in favor of the restricted model, probably due to an implicit regularization effect).

**Table 1:** Performance of depth-2 XGBoost prediction models with or without restricting gene-covariate interactions.

Phenotype	Metric	w/o restriction	with restriction
Height	*r* ^2^	0.666	0.666
eBMD	*r* ^2^	0.264	0.265
Hypothyroidism	AUC	0.799	0.799
Asthma	AUC	0.656	0.659
Psoriasis	AUC	0.725	0.725

**Pure covariate interactions**: The presence of these interactions can be tested by comparing XGBoost models of depth 1 and 2 on genotype data with covariates. In this case, we do observe a small increase of accuracy in depth-2 models for all phenotypes except height (see Fig 3).

To further confirm the nonlinear effects of covariates, we compare Snpnet and XGBoost models fitted on the exact same dataset. Table 2 shows performance of these models for common SNP sets selected either by XGBoost or GWAS. While for small number of covariates Snpnet generally outperforms or is on par with XGBoost, for 20+ covariates XGBoost consistently outperforms Snpnet, especially for hypothyroidism and asthma.

**Table 2:** Comparison of GWAS- and XGBoost-based (denoted XS) SNP selections.

	Metric	#(SNPs)	#(Cov)	Xgboost XS	Xgboost GWAS	Snpnet XS	Snpnet GWAS
eBMD	*r* ^2^	10K	27	**0.264**	0.254	0.263	0.251
Hypothyroidism	AUC	2K	20	**0.800**	0.794	0.793	0.782
Asthma	AUC	1K	20	**0.655**	0.645	0.632	0.615
Psoriasis	AUC	1K	20	**0.725**	0.713	0.717	0.713
Height	*r* ^2^	10K	2	0.666	0.636	**0.672**	0.640
eBMD	*r* ^2^	10K	2	0.230	0.219	**0.233**	0.221
Hypothyroidism	AUC	5K	2	0.770	0.766	**0.775**	0.769
Asthma	AUC	1K	2	**0.633**	0.617	0.622	0.604
Psoriasis	AUC	1K	2	0.689	0.695	**0.697**	0.690
Height	*r* ^2^	10K	1	0.629	0.591	**0.649**	0.617
eBMD	*r* ^2^	10K	1	0.207	0.201	**0.216**	0.205
Hypothyroidism	AUC	5K	1	0.749	0.713	**0.761**	0.760
Asthma	AUC	1K	1	**0.620**	0.598	0.612	0.590
Psoriasis	AUC	1K	1	0.687	0.686	**0.698**	0.693

### Analysis of XGBoost selection

Since SNP selection by XGBoost is our main technical innovation in this paper, we provide now a more in-depth analysis of its properties.

#### Detection of epistatic interactions

As already discussed, we have not achieved any improvement in prediction accuracy for our five UK Biobank phenotypes by exploiting potential gene-gene and gene-covariate interactions. A natural question is whether this is because such interactions are too weak or because our prediction pipeline is simply not capable of taking advantage of them.

**Fig 5:**
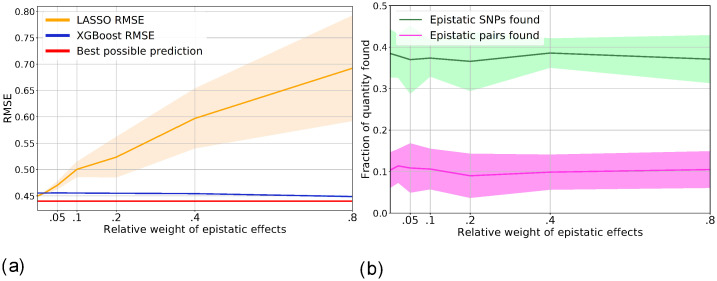
Evaluation of XGBoost prediction and selection performance on simulated data, using 100K samples, 1K SNPs, 100 linearly associated SNPs, 50 epistatic pairs. The weight of random noise is *w*_*ϵ*_ = 0.2, the total weight *w*_*ep*_ of epistatic effects ranges from 0 to 0.8, the total variance of linear effects equals 1 − *w*_*ep*_ − *w*_*ϵ*_, i.e. varies from 0.8 to 0. Shadows of lines have width of two standard deviations. **(a)** Prediction performance of Lasso and XGBoost when varying the relative weight of epistatic effects in the simulated phenotype. **(b)** Fraction of epistatic SNPs and epistatic SNP pairs found by XGBoost. An SNP is considered found when its importance is in the top 300 most important SNPs. A pair is considered found when both SNPs are in the top 300 most important SNPs.

To answer this question, we tested our pipeline on simulated phenotypes. We performed a number of simulations with both linear and epistatic effects and evaluated the detection power of XGBoost feature importance scores. Results are shown in Fig 5. We see that for simulated phenotypes, XGBoost catches most of the epistatic variance and consistently outperforms Lasso. Also, XGBoost is able to detect roughly 55% of epistatic SNPs. Therefore, we expect XGBoost to outperform Lasso on a phenotype with strong enough epistatic effects. Summarizing, we attribute the lack of improvement from using our nonlinear models on the real phenotypes to the lack of nonlinear information in the data rather than to deficiencies of the models.

#### Correlations between selected SNPs

**Fig 6:**
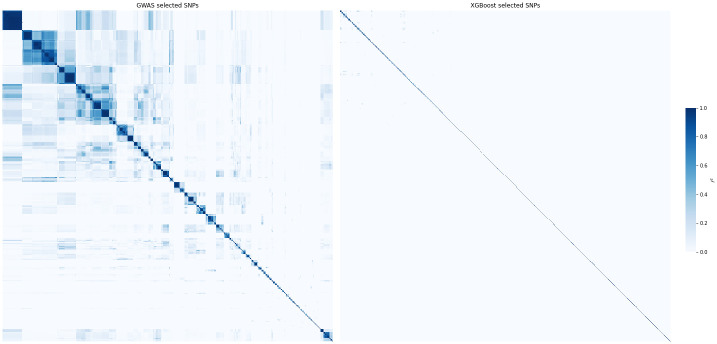
Clustered correlation heatmaps between top 1K SNPs selected by GWAS (left) and XGBoost (right) for asthma with 20 covariates, sex and PCA. GWAS selects SNPs by lowest p-value, XGBoost selects SNPs by highest importance score. The SNP sets in the left and right subfigure are different; the SNPs are sorted to produce clusters on the heatmaps. The color shows the squared correlation coefficient *r*^2^ between SNPs, estimated by plink 1.9. The average *r*^2^ is 0.0593 for GWAS and 0.0063 for XGBoost.

Since XGBoost-based SNP selection takes into account the already chosen SNPs when selecting the next ones, we expect the whole selected set of SNPs to be in an approximate linkage equilibrium. This hypothesis can be tested by comparing correlation between GWAS-selected SNPs with correlations between XGBoost-selected SNPs. The respective correlation matrices for asthma are shown in Fig 6. We see that SNPs selected by XGBoost are significantly less correlated: the mean squared correlation coefficient of the top 1K SNPs is 0.0593 for GWAS and 0.0063 for XGBoost.

#### GWAS vs XGBoost selection for the same number of SNPs

The lower correlation between SNPs selected by XGBoost compared to GWAS suggests that the former selection is more efficient in the sense of producing SNP subsets that are smaller while carrying a comparable amount of relevant information about the phenotype. To test this, we compared performance of these two SNP selection methods at fixed numbers of selected SNPs (Table 2). For each phenotype, the number of SNPs was chosen as the number after which the XGBoost model built on XGBoost-selected SNPs stops improving. In all cases, the dataset of XGBoost-selected SNPs allows to achieve a better accuracy compared to the dataset of GWAS-selected SNPs of the same size. GWAS starts to outperform XGBoost selection only when it selects at least twice the number of SNPs.

Our analysis also shows that XGBoost is better for the final prediction when the number of covariates is big, while Snpnet makes better use of SNP information (Table 2). This finding is consistent with our observation of exploitable nonlinearity present in covariates but not in genotype data.

**Fig 7:**
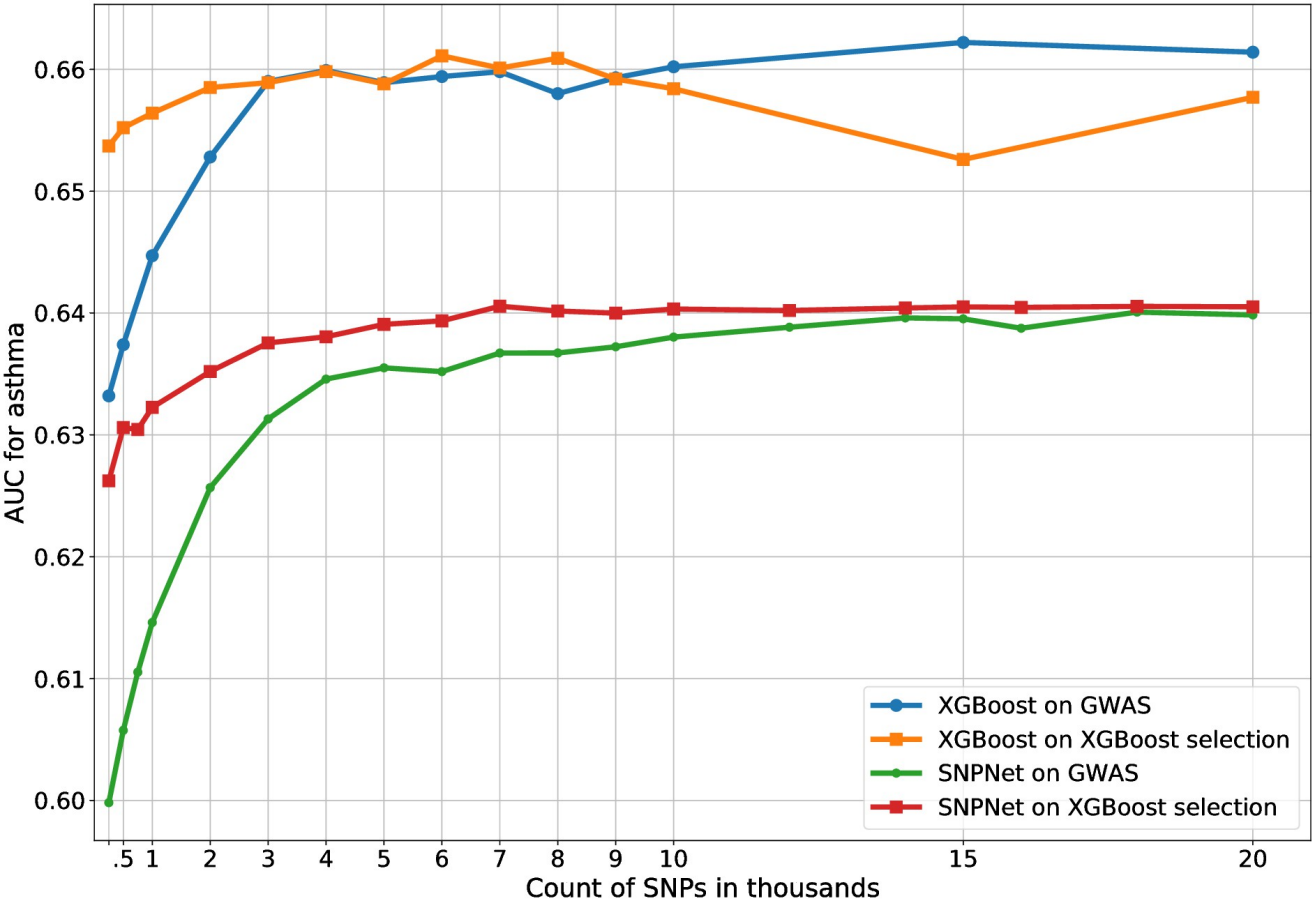
Dependence of the final model accuracy on the number of SNPs for four different combinations of selection and prediction methods for asthma. Both XGBoost selection and XGBoost prediction used depth-2 models. The features include 20 covariates, age, sex, and 10 principal components.

For a deeper analysis, in Fig 7 we show performance of the four combinations of the two selection methods (GWAS or XGBoost) with the two predictive models (XGBoost or Snpnet), for predicting asthma. The two GWAS-based pipelines show a similar pattern of worse performance on a small set of SNPs and plateauing after roughly 15K SNPs. The other two pipelines work much better on small sets of SNPs but saturate quicker, roughly at 5–7K SNPs. In agreement with results in Fig 3, the XGBoost prediction models are generally better than Snpnet for this phenotype (asthma) at a large number of covariates. This is, of course, not always the case for other phenotypes.

We observed similar results for XGBoost selection for all of the other phenotypes. It is probably less sensitive to SNPs with very small effects in comparison to GWAS. Therefore, we recommend selecting SNPs using XGBoost and then training a prediction model with 5K SNPs as features and *max*_*depth* = 2, *alpha* = 15 as starting set of hyperparameters. For other datasets, we recommend training a bunch of XGBoost models using different counts of SNPs and choose a saturation threshold after. In our experiments on GWAS-selected SNPs for height, XGBoost prediction model gives diminishing returns, but does not completely saturates using 20–50K SNPs. Similar behaviour was observed for predicting height with Lasso in [34]. However, XGBoost with >300K samples and >20K features cannot work on GPU with 16GB VRAM and is extremely slow on CPU, taking more than one day to train on 24 CPU cores with 160GB RAM. We recommend training XGBoost prediction model with more than 20K features only if one have more than 160GB RAM and a lot of computing power.

#### Effects of SNP pre-ordering

In contrast to GWAS selection, XGBoost selection depends on the order in which the SNPs are processed. We checked the importance of this order by running selection on the eBMD phenotype with the natural forward or backward genome order as well as with randomly shuffled windows. On the validation data set, we obtained *r*^2^ = 0.2771 for the forward pass, *r*^2^ = 0.2757 for the backward pass, and *r*^2^ = 0.2716 for the shuffled dataset. The forward+backward ensemble has *r*^2^ = 0.2807, and adding shuffled predictions negligibly improves the results (to *r*^2^ = 0.2809). This shows that the SNP order and local effects do contribute to the performance of XGBoost selection, but their influence is fairly limited. Nevertheless, the models resulting from different orderings are sufficiently diverse to allow some accuracy gain to be achieved by their ensembling.

#### Existing tree-based SNP selection models

The application of tree-based models to SNP selection is not widely explored but there are a few papers about it in the literature. The first one uses the gradient boosting framework LightGBM as a substitute for GWAS, which they call LightGWAS [38]. The authors apply LightGBM to the whole genotype matrix from a simulated dataset with 10K SNPs and select putative causal SNPs by feature importances derived from LightGBM. Our approach differs from theirs in several ways. First, applying a tree-based method directly to a real-world genotype matrix with > 500K SNPs is unfeasible. We propose window-based boosting to overcome this limitation. Second, we test our method on UK Biobank data and show that it works with large-scale real world data. Another difference is that we test three different hypotheses about the qualitative aspects of the selected features sets. We found that XGBoost selects a set of uncorrelated SNPs which cannot be obtained by GWAS or even LASSO after GWAS. Also, XGBoost can utilize nonlinearity in covariates. Finally, the authors assess the performance of LightGWAS using logistic regression which is a linear method, in contrast with our more comprehensive analysis comparing Snpnet and XGBoost predictive models on feature sets selected by XGBoost.

Another study in the domain of tree-based SNP selection concerns the selection of interacting SNPs by XGBoost applied to Finnish breast cancer data [39]. The authors propose using XGBoost for the initial SNP selection and a ranking algorithm to select the most important putative interactions. The key differences mentioned in the previous paragraph about window-based boosting, applying to biobank-scale data and comprehensive analysis of predictive models are also applicable here. Another difference is that we explicitly show that putative SNP-SNP and even SNP-environment interactions do not play an important role for the phenotype predictions, and that the most prominent feature selection methods should focus on genome-wide SNP selection instead of single SNP ones such as GWAS.

The most recent study is about searching for SNP-SNP and SNP-environment interactions using XGBoost and SHAP (SHapley Additive exPlanations) values in UK Biobank data for obesity prediction [40]. The authors selected 50K individuals as a ranking dataset, divided it into several SNP subsets with low mutual correlation and built a number of XGBoost models for every subset. Then, SHAP feature importance values and SHAP feature interaction values were calculated and used for the final analysis. In the end, the PR-AUC metric of the final obesity prediction model showed an improvement of 0.5–0.7%. The key differences with our approach are as follows. First, we propose a single-pass XGBoost screening, which takes only 2 hours for the whole UK Biobank genotype dataset. Second, we show that possible SNP-SNP and SNP-environment interactions do not significantly improve the final predictions and that improvement comes from using a set of SNPs different from those selected by GWAS along with environment feature interactions. Finally, we built ensemble prediction models and showed a significant improvement for a number of phenotypes, thus more clearly displaying the practical utility of our approach.

In conclusion, the uniqueness of our approach is that is a very fast, single-pass XGBoost SNP screening which is easily applicable to the biobank-scale data. SNPs selected by XGBoost significantly improve ensemble prediction models.

## Conclusion

### Nonlinearity

Our results for five UK Biobank phenotypes show that gene-gene and gene-covariate interactions in the data are not strong enough to be exploitable by state-of-the-art nonlinear predictive models so as to allow them to outperform best linear models. This conclusion is based on our comparison of various linear and nonlinear models, with and without cross terms. This conclusion is also confirmed by our study of simulated phenotypes with epistatic interactions on the same genotype data: for such phenotypes, prediction accuracy is consistently improved by using nonlinear models. At the same time, we also observe this improvement for real phenotypes if sufficiently many covariates are included as features for predictions. This shows that the covariates contain more useful nonlinear information than SNP-covariate or pure SNP associations.

### SNP selection

We have proposed a new approach to SNP selection, using a sequential construction of XGBoost models on a series of SNP windows and a simultaneous SNP importance estimation. Compared to the conventional GWAS-based SNP selection, our method creates a more informative and less correlated set of SNPs. For small sets of selected SNPs, models built using our selection pipeline consistently outperform those built using the standard GWAS pipeline. Another essential aspect of our approach is its dependence on the order of features during selection: forward and backward passes lead to different selected sets and hence models; we have shown that these models can be sufficiently diverse for their ensembling to yield even better models.

### Most accurate models

For all phenotypes, the most accurate models are obtained by ensembling indepedently trained models (Snpnet, XGBoost). We observe ensembles to be consistently more accurate than individual models, even for those phenotypes (height, eBMD, asthma) where one of the methods significantly outperforms the others. On the whole, ensembling of the state-of-the-art linear (Snpnet) as well as nonlinear (XGBoost depth-2) models seems to boost the overall accuracy by fully retaining the advantages of the constituent models.

Regarding individual (non-ensembled) models, we found that for one of the considered phenotypes (asthma), our nonlinear XGBoost depth-2 model significantly outperformed the state-of-the-art linear model (Snpnet).

## Supporting information

S1 File (TXT)

S1 Fig Estimates of the standard deviations of metrics. Estimates of the standard deviations *σ*_*N*_ of accuracy characteristics (*r*^2^, ROC AUC) for the five phenotypes as functions of the parameter *M* (see S1 Appendix, Eq. (5)).
(TIF)

S1 Table Numerical values of standard error estimates of the metrics for all considered phenotypes. (PDF)

S2 Table Prediction accuracy metrics for XGBoost and Snpnet models with different sets of covariates. (PDF)

S3 Table Metrics for our most accurate predictions obtained by ensembling different models for each phenotype. (PDF)

S4 Table Lists of additional covariates included in our models for each phenotype. (XLSX)

S1 Appendix Details of our methods for estimating standard errors and experiments with simulated phenotypes. (PDF)
