# Supplementary figures and images for: Human genotype-to-phenotype predictions: Boosting accuracy with nonlinear models
Source: PLoS One. 2022 Aug 31;17(8):e0273293. doi: 10.1371/journal.pone.0273293 (PMC9432766; doi:10.1371/journal.pone.0273293)

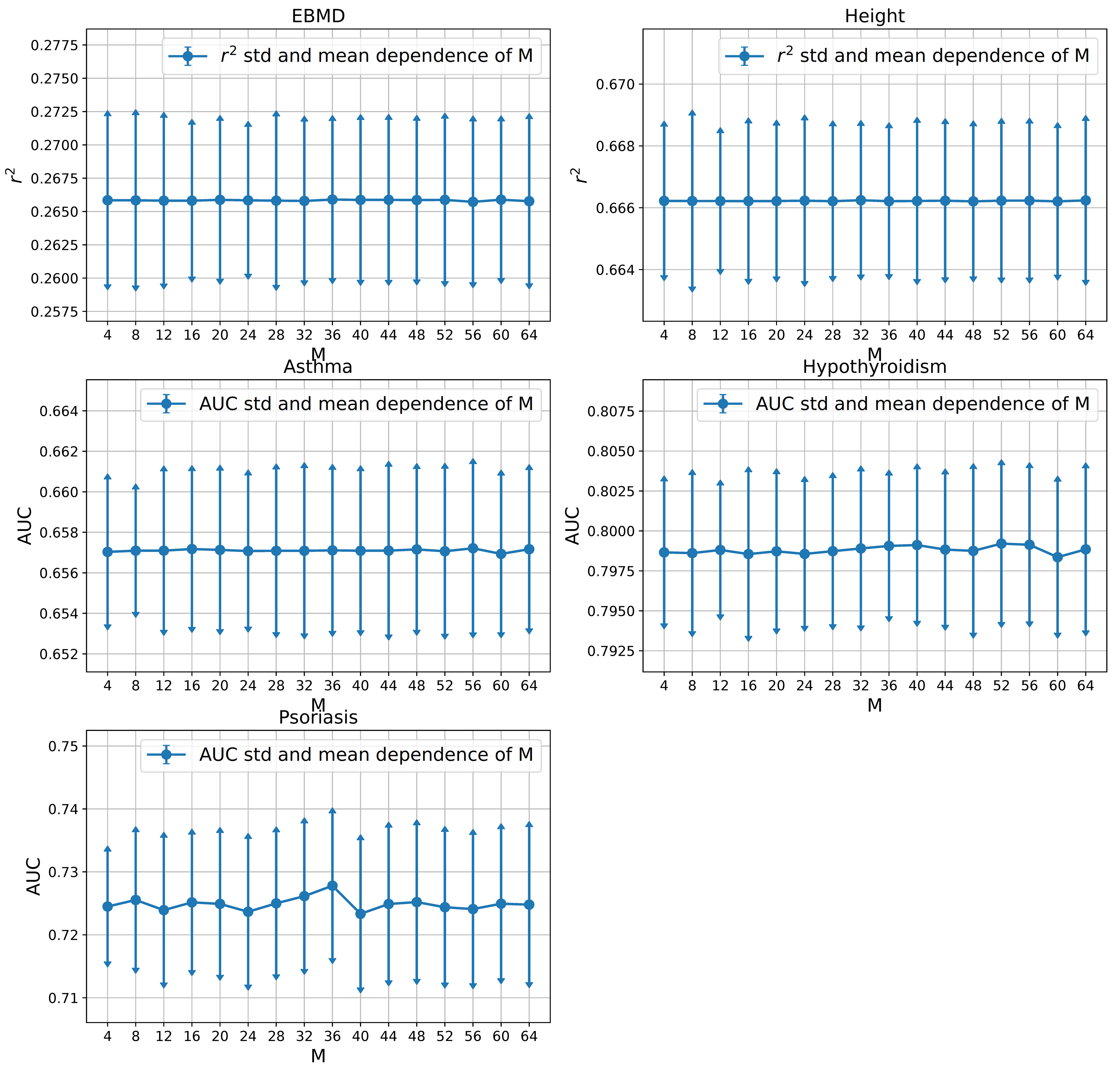

Supplement: S1 Fig — Estimates of the standard deviations σN of accuracy characteristics (r2, ROC AUC) for the five phenotypes as functions of the parameter M (see S1 Appendix, Eq. (5)). (TIF) [file pone.0273293.s002.tif]
