# Supplementary material for: Human genotype-to-phenotype predictions: Boosting accuracy with nonlinear models
Source: PLoS One. 2022 Aug 31;17(8):e0273293. doi: 10.1371/journal.pone.0273293 (PMC9432766; doi:10.1371/journal.pone.0273293)
Supplement: S1 Table — (PDF) [file pone.0273293.s003.pdf]

Standard errors for the accuracy metrics. The columns σ_{AUC} and σ_N correspond to the two different estimation methods.

	Metric	σ_{AUC} (Eq. (7), S1 Appendix)	σ_N (Eq. (5), S1 Appendix)
Height	r^2	-	0.00239
eBMD	r^2	-	0.00623
Asthma	AUC	0.00393	0.00422
Hypothyroidism	AUC	0.00474	0.00490
Psoriasis	AUC	0.01140	0.01226
