# Supplementary material for: Human genotype-to-phenotype predictions: Boosting accuracy with nonlinear models
Source: PLoS One. 2022 Aug 31;17(8):e0273293. doi: 10.1371/journal.pone.0273293 (PMC9432766; doi:10.1371/journal.pone.0273293)
Supplement: S2 Table — (PDF) [file pone.0273293.s004.pdf]

Accuracy of XGBoost and SNPnet models constructed for the 5 UK Biobank phenotypes. The SNPs were selected using XGBoost selection for the XGBoost models, and GWAS for SNPnet. The column #SNP shows the number of selected SNP's for all methods except SNPnet with unlimited SNPs, for which this number is indicated separately.

	Metric	#SNP	Covariates	XGBoost depth 1	Xgboost depth 2	SNPnet lim. SNPs	SNPnet unlim. SNPs
eBMD	r^2	10K	27 + PCA	0.258	0.264	0.251	0.280 (50K)
Hypothyroidism	AUC	2K	20 + PCA	0.793	0.800	0.782	0.800 (20K)
Asthma	AUC	1K	20 + PCA	0.651	0.655	0.615	0.643 (50K)
Psoriasis	AUC	5K	20 + PCA	0.720	0.725	0.713	0.726 (10K)
Height	r^2	10K	2 + PCA	0.666	0.666	0.640	0.686 (50K)
eBMD	r^2	10K	2 + PCA	0.223	0.230	0.214	0.249 (50K)
Hypothyroidism	AUC	5K	2 + PCA	0.770	0.769	0.769	0.775 (50K)
Asthma	AUC	5K	2 + PCA	0.633	0.633	0.627	0.635 (50K)
Psoriasis	AUC	5K	2 + PCA	0.703	0.704	0.706	0.709 (10K)
Height	r^2	10K	1	0.631	0.629	0.617	0.663 (50K)
eBMD	r^2	10K	1	0.208	0.207	0.205	0.233 (50K)
Hypothyroidism	AUC	5K	1	0.751	0.749	0.742	0.767 (20K)
Asthma	AUC	5K	1	0.621	0.620	0.617	0.626 (50K)
Psoriasis	AUC	5K	1	0.700	0.705	0.707	0.711 (20K)
