# Supplementary material for: Human genotype-to-phenotype predictions: Boosting accuracy with nonlinear models
Source: PLoS One. 2022 Aug 31;17(8):e0273293. doi: 10.1371/journal.pone.0273293 (PMC9432766; doi:10.1371/journal.pone.0273293)
Supplement: S3 Table — (PDF) [file pone.0273293.s005.pdf]

Comparison of best prediction methods. The values in parentheses show the number of pre-selected SNPs in the individual models; these numbers were chosen as providing the best performance. The XGBoost Ensembles consist of two XGBoost models trained on two different sets of SNPs, selected by either forward of backward pass over the genome. The XGB+Snpnet Ensemble and Stacking refer, respectively, to the unweighted or weighted average of the XGBoost forward, XGBoost backward and Snpnet.

Phenotype	Metric	Covariates	XGBoost Ensemble	Snpnet unlim. SNPs	XGB + Snpnet Ensemble	XGB + Snpnet Stacking
Height	r^2	2	0.671 (10K)	0.686 (50K)	0.685	0.690
eBMD	r^2	27	0.269 (10K)	0.280 (50K)	0.286	0.290
Hypothyroidism	AUC	20	0.799 (2K)	0.800 (20K)	0.809	0.810
Asthma	AUC	20	0.658 (1K)	0.643 (50K)	0.672	0.672
Psoriasis	AUC	20	0.724 (5K)	0.726 (10K)	0.730	0.731
