# Supplementary material for: Human genotype-to-phenotype predictions: Boosting accuracy with nonlinear models
Source: PLoS One. 2022 Aug 31;17(8):e0273293. doi: 10.1371/journal.pone.0273293 (PMC9432766; doi:10.1371/journal.pone.0273293)
Supplement: S1 Appendix — (PDF) [file pone.0273293.s007.pdf]

S1 Methods: additional details

S1.1 Estimation of standard errors

We estimate the standard error of accuracy metrics r^2 and ROC AUC of our predictive models using two approaches that produce similar results. The first approach is applicable to both metrics and is based on subsampling and a natural scaling assumption. The second approach is only applicable to ROC AUC as it uses the particular form of this statistic.

S1.1.1 A subsampling-based estimate

Let β be one of the accuracy metrics (i.e., r^2 or ROC AUC). Let β_* be its true population value, and let β_N be its estimate obtained using a random N-element sample. The estimate β_N is a random variable converging to the deterministic value β_* in the limit $N \to \infty$. We will assume that the estimate β_N is unbiased up to $O(\frac{1}{N})$:

$$\mathsf{E}\beta_N = \beta_* + O(\tfrac{1}{N}), \tag{1}$$

and its variance scales as $\sim \frac{\sigma^2}{N}$ with some $\sigma^2 > 0$:

$$\mathsf{E}(\beta_N - \mathsf{E}\beta_N)^2 = \mathsf{E}(\beta_N - \beta_*)^2 + O(\tfrac{1}{N^2}) = \tfrac{\sigma^2}{N}(1 + o(1)). \tag{2}$$

Under these assumptions, for large N, we can take the standard error for β_N to be

$$\sigma_N = \tfrac{\sigma}{\sqrt{N}}. \tag{3}$$

It remains to estimate σ^2. This can be done in the usual way, by considering M independent samples $D_1, \ldots D_M$ of size $\widetilde{N}$. Denote the respective values of the statistic β by $\beta_{\widetilde{N},1}, \ldots \beta_{\widetilde{N},M}$. Using the standard unbiased estimate of the variance and Eq. (2), and assuming that both $\widetilde{N}, M$ are large, we can write

$$\sigma^2 \approx \tfrac{\widetilde{N}}{M-1} \sum_{k=1}^{M} (\beta_{\widetilde{N},k} - \overline{\beta_{\widetilde{N}}})^2, \tag{4}$$

where $\overline{\beta_{\widetilde{N}}} = \frac{1}{M}\sum_{k=1}^{M} \beta_{\widetilde{N},k}$. In practice, we form the samples $D_1, \ldots D_M$ by randomly dividing the size-N sample into M equal parts, so that, in particular, $\widetilde{N} = N/M$. Combining Eq. (3) with Eq. (4), we conclude that we can estimate the standard error for a size-N sample by

$$\sigma_N \approx \Big(\tfrac{1}{M(M-1)} \sum_{k=1}^{M} (\beta_{\widetilde{N},k} - \overline{\beta_{\widetilde{N}}})^2\Big)^{1/2}. \tag{5}$$

Though this formula depends on M, the resulting estimate σ_N should be approximately independent of M. In Figure S1 we plot σ_N computed for all our phenotypes at various values of M, and indeed observe an approximate independence of M, confirming our methodology.

S1.1.2 Standard error for ROC AUC

Suppose that a "soft" binary classifier produces a numerical value $x_k \in \mathbb{R}$ for the k'th sample of class 0, and a value $y_l \in \mathbb{R}$ for the l'th sample of class 1. The ROC AUC of such a classifier is defined by

$$\beta = \frac{1}{n_0 n_1} \sum_{k=1}^{n_0} \sum_{s=1}^{n_1} \big(\mathbf{1}(x_k < y_l) + \tfrac{1}{2}\mathbf{1}(x_k = y_l)\big), \tag{6}$$

where n_0, n_1 are the numbers of samples in the classes 0 and 1.

Assuming that the samples x_k and y_l are randomly drawn from populations with a continuous distribution function, the variance of this classifier is (see [1, 2])

$$\sigma^2_{AUC} = \mathsf{E}(\beta - \beta_*)^2 = \frac{\beta_*(1-\beta_*) + (n_1 - 1)(\mathsf{P}(x < \min(y_1, y_2)) - \beta_*^2) + (n_0 - 1)(\mathsf{P}(\max(x_1, x_2) < y) - \beta_*^2)}{n_0 n_1}, \quad (7)$$

where $\beta_* = \mathsf{E}\beta$ is the population mean.

Assuming that n_0 and n_1 are large, the leading term of σ^2_{AUC} is

$$\sigma^2_{AUC} \approx \frac{\mathsf{P}(x < \min(y_1, y_2)) - \beta_*^2}{n_0} + \frac{\mathsf{P}(\max(x_1, x_2) < y) - \beta_*^2}{n_1}. \quad (8)$$

In particular, if x and y have the same distribution, then $\beta_* = \frac{1}{2}$ and $\mathsf{P}(x < \min(y_1, y_2)) = \mathsf{P}(\max(x_1, x_2) < y) = \frac{1}{3}$, so that

$$\sigma^2_{AUC} \approx \frac{n_0 + n_1}{12 n_0 n_1}. \quad (9)$$

In the general case, when x and y have different distributions, we can estimate the probabilities $\mathsf{P}(x < \min(y_1, y_2))$ and $\mathsf{P}(\max(x_1, x_2) < y)$ by sampling. Using additionally the estimated value β_* of ROC AUC, we can then obtain an estimate for σ^2_{AUC}.

In Table S1 we provide the standard errors estimated for our phenotypes by the two methods described in this and the previous section. In the case of ROC AUC the results are quite close, confirming the validity of our estimates.

S1.2 Simulated phenotypes: simulation details

A simulated phenotype is modelled as a weighted linear combination of linear effects $\mathbf{y}_l$, pairwise epistatic effects $\mathbf{y}_{ep}$ and random noise $\mathbf{y}_\epsilon$:

$$\mathbf{y} = w_l \mathbf{y}_l + w_{ep} \mathbf{y}_{ep} + w_\epsilon \mathbf{y}_\epsilon.$$

Here, $\mathbf{y}$, $\mathbf{y}_l$, $\mathbf{y}_{ep}$, $\mathbf{y}_\epsilon$ are vectors with N components corresponding to different simulated samples. The weights w_l, w_{ep}, w_ϵ are sample-independent and sum to 1. The values of the weights depend on the simulation run. For simulation experiments that are presented in this paper we set $w_\epsilon = 0.2$ and vary w_{ep} from 0 to 0.8.

Linear effects $\mathbf{y}_l$ are generated by $\mathbf{y}_l = X\mathbf{f}_l$, where X is the $N \times M$ genotype matrix, with M the number of SNPs and $\mathbf{f}_l$ the M-dimensional column vector of individual SNP effects. In all our experiments $M = 1000$ and $N = 100000$. Importantly, $\mathbf{f}_l$ has only $0.1M = 100$ non-zero entries which are distributed normally with zero mean and unit variance. This is done to mimic the sparsity of real-world genotype-phenotype interaction. Linear effects are scaled inversely by MAF (minor allele frequency) of the corresponding SNP to reflect the fact that rarer SNPs typically have larger effect sizes [3]. Non-zero entries of $\mathbf{f}_l$ are clipped to the interval $[-5, 5]$.

To generate pairwise epistatic effects, we randomly select 25 pairs of SNPs from the SNPs already associated linearly with the phenotype, and the same number from the SNPs not associated with the phenotype. For each pair $i = (i_1, i_2)$ of SNPs we generate a random epistatic effect size $f_{ep,i} \sim \mathcal{N}(0, 1)$ and randomly select a pair of SNP minor allele counts c_1, c_2, i.e. one value from the set $\{0, 1, 2\}$ for each SNP in the pair. Then, the epistatic effect for pair i and sample j is calculated by

$$y^{ij}_{ep} = \begin{cases} f_{ep,i}, & \text{if } X_{j,i_1} = c_1 \text{ and } X_{j,i_2} = c_2, \\ 0, & \text{otherwise.} \end{cases} \quad (10)$$

Here, X_{j,i_1} and X_{j,i_2} are the minor allele counts of the first and second SNP in the i'th pair, respectively. The total value of epistatic effects for sample j is

$$y^j_{ep} = \sum_{i=1}^{50} y^{ij}_{ep}.$$

In contrast to the linear effects, the epistatic effects are not scaled inversely with MAF.

References

[1] Henry B Mann and Donald R Whitney. On a test of whether one of two random variables is stochastically larger than the other. *The annals of mathematical statistics*, pages 50–60, 1947.

[2] Corinna Cortes and Mehryar Mohri. Confidence intervals for the area under the roc curve. In *Advances in Neural Information Processing Systems (NIPS 2004)*, Vancouver, Canada, 2005.

[3] Ju-Hyun Park, Mitchell H. Gail, Clarice R. Weinberg, Raymond J. Carroll, Charles C. Chung, Zhaoming Wang, Stephen J. Chanock, Joseph F. Fraumeni, and Nilanjan Chatterjee. Distribution of allele frequencies and effect sizes and their interrelationships for common genetic susceptibility variants. *Proceedings of the National Academy of Sciences*, 108(44):18026–18031, 2011.
